# Reviewed article: Bastos MES, Nascimento AJF, Soares MTF, Spinelli MBAS, Low ST. Knowledge, attitudes and practices of pregnant women for preventing risks and accidents with their newborns: cross-sectional study, Recife, 2024. Epidemiol Serv Saude. 2026:35;e20240822

**DOI:** 10.1590/S2237-96222026v35e20240822.a

**Published:** 2025-12-08

**Authors:** Roberta Costa

**Affiliations:** 1 Universidade Federal de Santa Catarina, Florianopolis, SC, Brazil

## Peer review A

## Questionnaire

Does the manuscript contain new and significant information to justify publication? Yes

Does the Abstract (Summary) clearly and accurately describe the content of the article? Yes

Is the problem significant and concisely stated? Yes

Are the methods described comprehensively? No

Are the interpretations and conclusions justified by the results? Yes

Is adequate reference made to other work in the field? Yes

## Manuscript Structure

Length of article is: Adequate

Number of tables is: Adequate

Number of figures is: Adequate

Please state any conflict(s) of interest that you have in relation to the review of this paper. None

Interest: Good

Quality: Average

Originality: Good

Overall: Average

Recommendation: Major Revision

## Comments

O texto apresenta uma temática relevante e importante para saúde pública. Os autores apresentam uma contextualização adequada do tema, entretanto sugiro aprofundar a justificativa e relevância do estudo. Em relação a método do estudo, necessita de maior detalhamento em diferentes aspectos, especialmente em relação a coleta de dados. Sugiro utilizar o checklist STROBE para guiar a descrição. Rever a forma de apresentação dos resultados, poruqe muitas vezes o texto está repetindo os dados das tabelas. Necessita aprofundar a discussão com os achados do estudo. Deixo em anexo alguns comentários para elucidar minhas sugestões.

